# Clinical Characteristics and Outcomes of Acute Ischemic Stroke in Patients with Type 2 Diabetes: A Single-Center, Retrospective Study in Southern China

**DOI:** 10.1155/2021/5517228

**Published:** 2021-06-14

**Authors:** Minrui Chen, Weiliang Luo, Jiming Li, Kaiyi Cao, Xiaohui Li, Huihong Huang, Yan He

**Affiliations:** ^1^Department of the First Clinical Medicine, Guangdong Medical University, Zhanjiang 524023, Guangdong, China; ^2^Department of Neurology, Huizhou Hospital Affiliated to Guangdong Medical University Huizhou Municipal Central Hospital, Huizhou 516001, Guangdong, China

## Abstract

**Objective:**

To explore the associations between type 2 diabetes mellitus (DM) and stroke by evaluating the clinical risk factors, characteristics, and outcomes of acute ischemic stroke (AIS) patients with and without type 2 DM.

**Methods:**

A total of 1,156 AIS patients (including 410 with type 2 DM (AIS-DM group)) and 746 without type 2 DM (AIS-NDM group)) were included. Patients' demographics, auxiliary examinations, clinical manifestations, and treatment outcomes were recorded and analyzed.

**Results:**

Among the included AIS patients, 35.46% had type 2 DM. The AIS-DM group had less males (59.76% versus 70.64%), less smokers (33.90% versus 41.96%), more patients with hypertension (72.93% versus 63.94%; *p*=0.002), higher triglyceride levels (42.93% versus 25.08%; *p* ≤ 0.01), and lower total cholesterol (147.06 mg/dl versus 175.31 mg/dl) than the AIS-NDM group. The proportion of patients with large artery atherosclerosis (LAA) in the AIS-DM group was lower (77.56% versus 85.92%; *p* < 0.05) than that in the AIS-NDM group, and the proportion of patients with small arterial occlusions (SAO) in the AIS-DM group was higher (27.07% versus 13.67%; *p* < 0.05) than that in the AIS-NDM group. The mean National Institutes of Health Stroke Scale (NIHSS) score at admission in the AIS-DM group was lower than that in the AIS-NDM group (4.39 versus 5.00; *p*=0.008), but there was no significant difference in the NIHSS score or the modified Rankin Scale score between the two groups at discharge. A total of 85 AIS patients underwent intravenous thrombolysis treatment with recombinant tissue plasminogen activator (rtPA). The door-to-needle time (DNT) did not differ significantly between the groups (49.39 ± 30.40 min versus 44.25 ± 15.24 min; *p*=0.433). In addition, there were no significant differences in the baseline NIHSS score, 7-day NIHSS score, and mRS score at discharge between the groups. After intravenous thrombolysis with rtPA, the AIS-NDM group had better recovery (44.30% versus 29.20%; *p*=0.017) and a higher ratio of good treatment outcome at discharge (65.60% versus 54.20%; *p*=0.762).

**Conclusions:**

Type 2 DM is associated with AIS and its risk factors, such as dyslipidemia and hypertension. Patients in the AIS-DM group had less LAA and smaller arterial occlusions, and DM could exacerbate the short-term clinical outcomes in AIS patients.

## 1. Introduction

Diabetes mellitus (DM) is a common chronic metabolic disorder characterized by a high blood glucose level, which has prevalence of 8.8% in the general population worldwide [1]. In 2019, approximately 463 million people had DM globally [2]. In the last 20 years, the prevalence of DM has increased rapidly in China, with an annual growth rate of approximately 0.72%, ranking it first among Asian countries and much higher than that in the United States [2]. In China, type 2 DM, which is known as adult-onset diabetes, accounts for approximately 95% of all DM cases [3]. In addition, DM increases the risk of stroke 2–5 times, and >90% of DM-induced strokes are due to cerebral infarction [4, 5].

Stroke is an acute cerebrovascular disease, which includes three subtypes: ischemic stroke, hemorrhagic stroke, and subarachnoid hemorrhage [6, 7]. In 2015, there were 6.3 million stroke-caused deaths, which accounted for 11% of the total deaths worldwide [8]. Recently, stroke remained the leading cause of death in China [9]. Acute ischemic stroke (AIS) is the most common stroke and accounts for 60%–80% of all stroke cases [7]. The risk factors of AIS include DM, hypertension, smoking, alcohol abuse, poor diet, hyperlipidemia, cardiac disorders, and lack of physical activity or exercise [10–12]. In recent decades, many studies have linked DM and stroke through clinical and experimental investigations [13–15]. DM causes various micro- and macrovascular changes, which lead to major clinical complications that include stroke [16]. Hyperglycemia in DM patients is a significant risk factor for the occurrence of stroke. In addition, hyperglycemia might be associated with poorer clinical outcomes (e.g., recurrence and higher disability or mortality) in ischemic and hemorrhagic stroke [16]. Recent studies showed that approximately 30% of AIS patients had DM, which could cause poorer clinical outcomes compared with those experienced by AIS patients without DM [17]. Altemini et al. found that 94.3% of patients with AIS had DM [11]. According to domestic studies in China, the prevalence of DM in patients with AIS ranged from 27.0% to 45.8% [18, 19]. Of note, the incidence rate of DM varies considerably in different regions. In general, the incidence in the central region of China is lower than that in western China, and the rates in economically developed regions are higher than those in less developed regions. Southern China is an economically developed region of China and has a higher incidence of type 2 DM [5]. However, compared with other regions, southern China has the lowest incidence of stroke [19]. There are no reports on the clinical characteristics and prognosis of AIS in patients with DM in southern China, to the best of the authors' knowledge. Huizhou is a city with >5 million people in Guangdong Province, southern China, and belongs to the economically developed regions of China. In this study, the correlation between AIS and type 2 DM is investigated in southern China by conducting a retrospective analysis. The clinical features and prognosis of AIS between patients with and without DM in this region are compared.

## 2. Methods

### 2.1. Subjects

This study enrolled 1,156 AIS patients that had been admitted (0–7 days after onset) to the Department of Neurology, Municipal Central Hospital, Huizhou City, China, from January 1, 2019, to December 31, 2019. In 2019, the hospital was named the National Advanced Stroke Center in China. The diagnostic criteria of AIS agreed with the guidelines set by the Stroke Prevention Project Commission of the Chinese National Health Commission. These diagnostic criteria were consistent with the criteria set by the American Heart Association/Stroke Association in 2013 [6]. Classification of AIS subtypes (e.g., large artery atherosclerosis (LAA), cardioembolism, small vessel (small arterial) occlusion, stroke of other determined etiologies, and stroke of undetermined etiology) was performed using the Trial of Org 10172 in Acute Stroke Treatment (TOAST) [20]. Informed consent was obtained from all subjects or subjects' guardians. The research study protocol was reviewed and approved by the Scientific Research Ethics Committee of Municipal Central Hospital of Huizhou City (permit no.: kyll2020107).

Patients were classified as having type 2 DM if they self-reported type 2 DM, had a DM medical record, and used hypoglycemic medications (e.g., insulin or sulfonylureas). If the patient reported that they had type 2 DM but had not used (or only irregularly used) hypoglycemic medication or the patient did not know whether they had type 2 DM or not, the diagnosis was made based on the following criteria: (1) glycated hemoglobin (HbA1c) ≥6.5%, (2) fasting plasma glucose (FPG) ≥126 mg/dL, (3) 2-hour blood glucose ≥200 mg/dL on oral glucose tolerance test, or (4) random blood glucose (RPG) ≥200 mg/dL with typical symptoms of hyperglycemia or hyperglycemic crisis [21].

Patient baseline information that included gender, age, blood pressure, HbA1c, and fasting blood triglycerides (TG)/total cholesterol(TC)/low-density lipoprotein cholesterol (LDL-C) was recorded upon admission. The assessment of baseline risk factors included a history of cerebral infarction, hypertension, atrial fibrillation, smoking, alcohol consumption, coronary heart disease, and heart failure. Serum TG/TC/LDL-C levels were measured by enzymatic measurement (Cobas c702 analyzer, Roche Diagnostics GmbH, Germany). Serum HbA1c levels were measured using high-performance liquid chromatography (Variant II analyzer, Bio-Rad Diagnostics GmbH, USA).

For the definitions of hypertension, atrial fibrillation, smoking, and alcohol consumption, the definitions used in the stroke's epidemiological risk factor survey in mainland China since 2013 were adopted [17]. Hypertension was defined as self-reported high blood pressure (systolic blood pressure ≥140 mmHg or diastolic blood pressure ≥90 mmHg or both), use of antihypertensive medications, or recorded hypertension in the medical record. Atrial fibrillation was defined by a previous medical history of atrial fibrillation or a new clinical diagnosis based on an electrocardiogram. Coronary heart disease was defined by a previous medical history of myocardial infarction or angina pectoris or a new clinical diagnosis based on the cardiovascular events that were confirmed by a cardiologist during hospitalization. Heart failure was defined by a previous medical history of heart failure or a new clinical diagnosis based on clinical symptoms. Dyslipidemia was defined as abnormal blood lipids detected in a blood test during hospitalization, use of blood lipid-lowering medication, or recorded abnormal blood lipid levels in the medical record. The abnormal cut-off values of lipid profile were diagnosed based on the Guidelines for the Prevention and Treatment of Dyslipidemia in Adults in China: (1) TC ≥ 200 mg/dL, (2) TG ≥ 150 mg/dL, or (3) LDL-C  ≥ 130 mg/dL [22, 23]. Alcohol consumption referred to alcohol intake ≥1 time/week at any time. Smoking was defined as ≥1 instance of tobacco use/day.

Stroke-related neurologic deficits and impairments were measured by the National Institutes of Health Stroke Scale (NIHSS) [24]. Clinical outcomes of patients were assessed by the modified Rankin Scale (mRS) [25].

### 2.2. Statistical Analysis

SPSS 26.0 software was used for data analysis. The count data were expressed as frequency/percentage, and the Chi-squared test was used to compare the data between both groups. Measurement data were expressed as mean ± standard deviation (SD). A Student's *t*-test was used for data with a normal distribution, and a nonparametric test was used for data without normal distribution. An independent-sample *t*-test was used for the comparison of independent samples. A *p* value ≤ 0.05 was considered statistically significant.

## 3. Results

A total of 1,156 AIS patients including 410 patients (35.46%) with type 2 DM (AIS-DM group) and 746 patients (64.53%) without type 2 DM (AIS-NDM group) were enrolled in this study. The risk factors of both groups were compared, and several differences were observed (Table 1). The AIS-DM group had fewer males (59.76% versus 70.64%) and fewer smokers (33.90% versus 41.96%). There was no significant difference in those who received ≥1 lipid-lowering drug for ≥3 months before admission between the AIS-DM group and AIS-NDM group (14.88% versus 17.96%; *p*=0.190). Lower TC (147.06 mg/dL versus 175.31 mg/dL) was found in the AIS-DM group (all *p* < 0.05). The AIS-DM group showed significantly higher TG (42.93% versus 25.08%; *p* ≤ 0.01). In addition, the AIS-DM group had a higher percentage of patients with hypertension (72.93% versus 63.94%; *p*=0.002).

The percentage of patients with LAA in the AIS-DM group was lower than that in the AIS-NDM group (77.56% versus 85.92%; *p* < 0.05). However, the percentage of patients with infarction with small arterial occlusion (SAO) in the AIS-DM group was higher than that in the AIS-NDM group (27.07% versus 13.67%; *p* < 0.05). At admission, the mean NIHSS score of the AIS-DM group was lower than that of the AIS-NDM group (4.39 versus 5.00; *p*=0.008), but no significant differences in the NIHSS score and mRS score were observed between both groups at discharge (Table 2).

Among the patients included in this study, 85 (7.3%) AIS patients (24 in the AIS-DM group and 61 in the AIS-NDM group) underwent rtPA intravenous thrombolysis treatment. There was no significant difference in door-to-needle time (DNT) between the two groups (44.25 ± 15.24 min versus 49.39 ± 30.40 min; *p*=0.433). In addition, there were no significant differences in the baseline NIHSS score, 7-day NIHSS score, and mRS score at discharge between the two groups (Tables 3 and 4). After intravenous thrombolysis with rtPA, the AIS-NDM group had better recovery (e.g., 7-day NIHSS score decreased by 4 points; 44.30% versus 29.20%; *p*=0.017; Table 4) and a higher percentage of patients with a favorable outcome by using mRS score at discharge (65.60% versus 54.20%; *p*=0.762; Table 5).

## 4. Discussion

The clinical characteristics and treatment outcomes of 1,156 AIS patients from southern China were examined in this study. In total, 35.46% of AIS patients had type 2 DM, and the AIS-DM group had a significantly higher rate of hypertension and abnormal TG compared with the AIS-NDM group. In addition, the AIS-DM group had a higher percentage of patients with SAO cerebral infarction and poorer treatment outcomes (regardless of thrombolysis or nonthrombolysis treatment) at discharge, compared with the AIS-NDM group.

Although many studies demonstrated associations between stroke (including AIS) and DM [13–18], the results of this study could offer a valuable contribution to the management of AIS-DM patients for the following reasons. First, the prevalence rates of stroke or type 2 DM or both in China are different from those in many other countries [3, 26]. For instance, China reported a higher incidence of stroke patients compared to other countries globally, and stroke is the most common cause of death in China [26]. In addition, China has the largest population with diabetes (mainly type 2 DM) within a single country [4]. Therefore, this study reflected the real-world conditions for type 2 DM and AIS in China. Second, the prevalence rates of stroke and type 2 DM show significant geographic variations throughout China [3–5, 9, 12]; therefore, this study reflected the real-world situation for DM and AIS in southern China, which is a developed region in China. Third, some studies were carried out many years ago and might not represent the current situation in China, in particular in southern China, which has experienced very rapid economic growth during the last two decades. Fourth, the outcomes for stroke patients in China appear to be comparable with those in other high-income countries, and one reason might be that Chinese stroke patients tend to be younger [26]. Therefore, knowledge on the associations between DM and AIS might improve the country or region-specific prevention and management of AIS.

In this study, 35.46% of AIS patients had type 2 DM, and this percentage agreed with the data in some previous studies [16, 18, 19, 27]. However, the results of this study did not agree with some other reports. Recently, Guangdong Province (in which Huizhou is located) and Shandong Province published the results of epidemiological investigations into stroke [28–30]. AIS-DM patients in Guangdong accounted for 20.0% of all stroke patients and this percentage was 12.0% in Shandong. However, the incidence of stroke in Shandong (northern China) was approximately two times that in Guangdong (southern China). This was because the prevalence of stroke is related to other risk factors, such as hypertension, atrial fibrillation, smoking, drinking, air pollution, and elevated blood lipid levels [26]. In addition, the difference in the prevalence of stroke might be related to the differences between the traditional northern diet (a high intake of refined cereal products and salt due to salted vegetables) and the traditional southern diet (high intake of rice, fruit, and vegetables and a low intake of salt) in China [26].

In this study, the mean prevalence of DM in stroke patients which was reported in Huizhou was higher than that in Guangdong, which indicated that, within the same province, the prevalence of DM, stroke, and AIS-DM might be related to other factors, such as socioeconomic status and geographic location. Even in Guangdong, the economic development and growth rate of each city are different. For example, Huizhou is less developed than Shenzhen and Guangzhou but is more developed than other cities, such as Shanwei and Yunfu. Therefore, this study could provide local and general guidelines for the prevention and management of AIS patients where DM is relatively common and poorly controlled, especially in China [26].

Hypertension and DM are two major well-established risk factors for stroke [31]. The AIS-DM group had a higher percentage of hypertension cases compared to the AIS-NDM group (72.93% versus 63.94%), which corresponded to a previous report in China (which had an incidence of 77.7%) and another report in USA (which had an incidence of 71%) [32, 33]. Although it is well known that type 2 DM and hypertension are common comorbidities of AIS, the exact mechanism has not been established. Hyperinsulinemia, hyperglycemia, increased oxidative stress, and subclinical chronic system inflammation in type 2 DM damage the endothelial cells, reduce vascular reactivity, increase peripheral vascular resistance, cause arterial remodeling or stiffness, and lead to atherosclerosis [32]. All of these pathological or pathophysiological changes contribute to the subsequent development of hypertension. The coexistence of type 2 DM and hypertension is particularly destructive, because both conditions are closely related to cardiovascular diseases, stroke, renal disease, and diabetic retinopathy [32]. Therefore, the management of blood pressure could be combined with glucose-lowering medications to prevent and treat AIS.

Epidemiological data indicated that the prevalence of dyslipidemia in adults that lived in mainland China was 41.9%, and 17.7% had high TG, but only 8.8% were treated with medication [33]. In addition, the rate of achievement of standard blood lipid levels among patients that take medication was very low. A recent survey showed that, for Chinese patients with dyslipidemia that received ≥1 lipid-lowering drug for 3 months, the rate of achieving the standard blood lipid levels among DM patients was 35.3% [34]. In this study, compared with the AIS-DM group, there was no significant difference in the rate from receiving ≥1 lipid-lowering drug treatment for ≥3 months before admission between the two groups. Several factors that include adipocytokines, insulin deficiency or resistance, and hyperglycemia might contribute to abnormal lipid metabolism that is seen in type 2 DM patient [35]. This type of dyslipidemia might contribute to the occurrence of AIS, especially atherosclerotic subtypes [36–38]. Many epidemiological cohort studies demonstrated a direct association between cholesterol levels and AIS [38]. Although some studies did not observe associations between higher TG and stroke [39, 40], in this study, the AIS-DM patients had higher TG levels than the AIS-NDM patients, which agreed with previously published results [40, 41]. Increased TG might lead to elevated lipoprotein remnant cholesterol, which might penetrate the artery walls, deliver lipid (cholesterol) into the intima layer, and eventually lead to atherosclerosis [40]. Therefore, the management of blood lipid levels and the manipulation of lipid metabolism in type 2 DM patients might be beneficial for the prevention and treatment of AIS, especially in China, where dyslipidemia is relatively common (34%) and often poorly controlled [26].

Lacunar infarct, the most frequent subtype of AIS, results from the occlusion of small arteries that supply the deep structures of the brain [42]. Overall, >90% of stroke patients with DM have cerebral infarction (mostly lacunar infarction), and DM is regarded as a major modifiable risk factor for lacunar infarct [43]. In this study, the percentage of patients with SAO in the AIS-DM group was significantly higher than that in the AIS-NDM group (27.07% versus 13.67%), which agreed with previous reports [43, 44]. One explanation could be that atherosclerosis (caused by type 2 DM) at the ostium of the perforating arteries of the brain might lead to lacunar infarct [43]. The NIHSS score at admission was lower in the AIS-DM group than in the AIS-NDM group. However, there were no differences in NIHSS scores, treatment outcomes (mRS score: 0–2), and mortality between the two groups at discharge, which agrees with some previous studies [43, 45].

Intravenous thrombolysis with rtPA is a specific and effective treatment for AIS. In this study, 85 AIS patients underwent rtPA intravenous thrombolysis. There was no statistical difference in the DNT time between both groups. However, the treatment was less effective in the AIS-DM group, because there were fewer patients with a decreased NIHSS score (≥4) after 7 days of rtPA treatment or with a mRS score of 0–2 at discharge. The worst outcome of rtPA intravenous thrombolysis in AIS-DM patients agreed with the results of previous reports [46, 47].

There are some limitations to this study. First, because this study was a single-center, retrospective study and the subject size was limited, selection bias was inevitable. Second, because the effectiveness of treatment was observed during hospitalization and at discharge, the long-term outcomes of the AIS patients were not evaluated. Third, for patients with type 2 DM, the duration and types of treatment or medications were not recorded, which might affect the evaluation of the data.

In conclusion, this study provided significant knowledge on the association between AIS and type 2 DM in the population of southern China. These results could facilitate the country or region-specific prevention and management of AIS by promoting reductions in modifiable risk factors.

## Figures and Tables

**Table 1:** Clinical risk factors of study participants.

Clinical risk factors	NDM group (*n* = 746)	DM group (*n* = 410)	Total (*n* = 1156)	*p* value
Gender (male) (*n*, %)	527 (70.64%)	245 (59.76%)	772 (66.78%)	≤0.010^*∗*^
Age (mean ± SD; years)	64.85 ± 12.71	64.03 ± 10.48	64.56 ± 11.97	0.164
History of AIS (*n*, %)	165 (22.12%)	78 (19.02%)	243 (21.02%)	0.228
Smoking (*n*, %)	313 (41.96%)	139 (33.90%)	452 (39.10%)	0.008^*∗*^
Drinking (*n*, %)	105 (14.13%)	56 (13.69%)	161 (13.93%)	0.929
Hypertension (*n*, %)	477 (63.94%)	299 (72.93%)	776 (67.13%)	0.002^*∗*^
Atrial fibrillation (*n*, %)	36 (4.83%)	25 (6.10%)	61 (5.28%)	0.409
Coronary heart disease (*n*, %)	50 (6.70%)	39 (9.51%)	89 (7.70%)	0.106
Heart failure (*n*, %)	34 (4.56%)	10 (2.44%)	44 (3.81%)	0.078
TG (≥150 mg/dL) (*n*, %)	187 (25.08%)	176 (42.93%)	363 (31.40%)	≤0.010^*∗*^
TG (mean ± SD; mg/dL)	148.85 ± 518.22	183.40 ± 202.01	160.37 ± 433.25	≤0.010^*∗*^
TC (≥200 mg/dL) (*n*, %)	209 (28.02%)	103 (25.12%)	312 (26.99%)	0.300
TC (mean ± SD; mg/dL)	175.31 ± 49.03	147.06 ± 81.08	165.25 ± 63.70	≤0.010^*∗*^
LDL-C (≥130 mg/dL) (*n*, %)	313 (41.96%)	160 (39.02%)	473 (40.92%)	0.349
LDL-C (mean ± SD; mg/dL)	114.55 ± 53.83	101.01 ± 63.24	109.52 ± 57.66	0.005^*∗*^
Statins/fenofibrate use (*n*, %)	134 (17.96%)	61 (14.88%)	195 (16.87%)	0.190

^*∗*^ *p* < 0.05. SD: standard deviation; NDM: no diabetes mellitus; AIS: acute ischemic stroke; TG: triglycerides; TC: total cholesterol; LDL-C: low-density lipoprotein cholesterol.

**Table 2:** Classification of etiology and prognosis at discharge of the participants.

Clinical characteristics	NDM group (*n* = 746)	DM group (*n* = 410)	Total (*n* = 1156)	*p* value
SAO (*n*, %)	102 (13.67%)	111 (27.07%)	213 (18.43%)	≤0.010^*∗*^
LAA (*n*, %)	641 (85.92%)	318 (77.56%)	959 (82.96%)	≤0.010^*∗*^
Mean NIHSS (on admission)	5.00	4.39	4.78	0.008^*∗*^
Mean NIHSS (at discharge)	3.46	3.44	3.46	0.161
Favorable prognosis (mRS score: 0–2) (*n*, %) (at discharge)	527 (70.64%)	281 (68.54%)	808 (69.90%)	0.462

^*∗*^ *p* < 0.05. NDM: no diabetes mellitus; SAO: small-artery occlusion; LAA: large-artery atherosclerosis; NIHSS: National Institutes of Health Stroke Scale; mRS: modified Rankin Scale.

**Table 3:** Comparison of outcomes of rtPA intravenous thrombolysis of both groups.

Group	*n*	Baseline NIHSS score (on admission)	7-day NIHSS score	mRS score (at discharge)	DNT
NDM	61	9.13 ± 6.36	5.98 ± 8.49	1.98 ± 1.63	49.39 ± 30.40
DM	24	6.63 ± 4.29	5.88 ± 6.09	2.50 ± 1.53	44.25 ± 15.24
*t*	—	1.776	0.570	−1.338	0.789
*p* value	—	0.079	0.955	0.185	0.433

^*∗*^ *p* < 0.05. rtPA: recombinant tissue plasminogen activator; SD: standard deviation; NDM: no diabetes mellitus; NIHSS: National Institutes of Health Stroke Scale; mRS: modified Rankin Scale; DNT: door-to-needle time.

**Table 4:** Comparison of 7-day NIHSS scores after rtPA intravenous thrombolysis of both groups.

Group	*n*	7-day NIHSS score decreased by >4 points	7-day NIHSS score decreased by <4 points	Effective rate
NDM	61	27	34	0.443
DM	24	7	17	0.292
Χ^2^	—	—	—	5.324
*p* value	—	—	—	0.017^*∗*^

^*∗*^ *p* < 0.05. rtPA: recombinant tissue plasminogen activator; NIHSS: National Institutes of Health Stroke Scale; NDM: no diabetes mellitus.

**Table 5:** Comparison of mRS scores at discharge after rtPA thrombolytic therapy of both groups.

Group	*n*	mRS score: 0–2 (favorable prognosis)	mRS score: 3–6	Proportion (mRS score: 0–2)
NDM	61	40	21	0.656
DM	24	13	8	0.542
Χ^2^	—	—	—	0.092
*p* value	—	—	—	0.762

^*∗*^ *p* < 0.05. mRS: modified Rankin Scale; rtPA: recombinant tissue plasminogen activator; NDM: no diabetes mellitus.

## Data Availability

The datasets used, analyzed, or both during this study are available from the corresponding author upon reasonable request.
